# Be Ready for Emergencies

**Published:** 1917-02-03

**Authors:** 


### THE EDITOR'S LETTER-BOX.

### Be Ready for Emergencies.

By the CHAIRMAN OF THE LONDON HOSPITAL.

*To the Editor of* THE HOSPITAL.

SIR,—Many years ago—twenty-five, I think—the captain of the Millwall Football Team was engaged in a charity football match, the proceeds of which were to be given to the Poplar Hospital. His leg was broken; they got him on to a stretcher and started to take him to the London Hospital. "No, no!" he said, "I want to go to Poplar." So he was taken there, and was refused admission because "We are full up"! Such criminal folly as this refusal is enough to ruin a hospital. Good came of it after the righteous indignation of the Millwall Team had been appeased, because I often visited the captain, Cahill by name, in Gloucester Ward of the London Hospital, where he was taken, and there got to know Sister Gloucester, who was very soon afterwards appointed matron of the Poplar Hospital, and there Miss Bland has been ever since. But the hospital was considerably injured, and deserved to be. This is merely an introduction to what I want to say.

We, at Poplar, were caught unready to meet an emergency, and I learned a lesson, and have taken care, both at that hospital and the London, that we should never be caught again. *Every hospital should be prepared to meet any sudden call on it, never mind how great. The greater the better.* To do this it is necessary to purchase and store a large number of extra bedsteads, and to keep the mattresses aired by letting the nurses sleep on two mattresses, or by keeping them in a properly warmed linen-room. It is not a bad record for Poplar, with only 100 beds, to have been able to fix up "somewhere" thirty extra beds to meet the hideous need of the late explosion. At the London 100 extra beds were put up in an hour from those in store. In the first year of the war we at the London were suddenly asked on a Sunday by telephone whether we could take in 100 wounded "just arriving at Waterloo Station"—the first to arrive. Nearly 200 came, but our store of extra bedsteads was there to meet the need.

May I venture, therefore, to urge on all hospitals to be similarly prepared against an evil day when they may be sorely tried, and, I trust, not found wanting?

Many are so prepared, I know, but I also know that all are not. Who can tell how great the call on the voluntary hospitals may not be on "the Day" when it comes?—Yours truly, KNUTSFORD.

Kneesworth Hall, Royston, Herts.

*** We welcome the above reply to our letter to the Poplar Hospital. An account of the part played by the Seamen's Hospital will be found in our current issue, and we hope to publish others in justice to the best-administered voluntary hospitals which have long pursued the practice of being ready for emergencies.—ED. THE HOSPITAL.

